# The impact of health service variables on healthcare access in a low resourced urban setting in the Western Cape, South Africa

**DOI:** 10.4102/phcfm.v7i1.820

**Published:** 2015-06-19

**Authors:** Elsje Scheffler, Surona Visagie, Marguerite Schneider

**Affiliations:** 1 Centre for Rehabilitation Studies, Stellenbosch University, South Africa; 2 Psychology Department, Stellenbosch University, South Africa; 3 Centre for Public Mental Health, Department of Psychiatry and Mental Health, University of Cape Town, South Africa

## Abstract

**Background:**

Health care access is complex and multi-faceted and, as a basic right, equitable access and services should be available to all user groups.

**Objectives:**

The aim of this article is to explore how service delivery impacts on access to healthcare for vulnerable groups in an urban primary health care setting in South Africa.

**Methods:**

A descriptive qualitative study design was used. Data were collected through semi-structured interviews with purposively sampled participants and analysed through thematic content analysis.

**Results:**

Service delivery factors are presented against five dimensions of access according to the ACCESS Framework. From a supplier perspective, the organisation of care in the study setting resulted in available, accessible, affordable and adequate services as measured against the District Health System policies and guidelines. However, service providers experienced significant barriers in provision of services, which impacted on the quality of care, resulting in poor client and provider satisfaction and ultimately compromising acceptability of service delivery. Although users found services to be accessible, the organisation of services presented them with challenges in the domains of availability, affordability and adequacy, resulting in unmet needs, low levels of satisfaction and loss of trust. These challenges fuelled perceptions of unacceptable services.

**Conclusion:**

Well developed systems and organisation of services can create accessible, affordable and available primary healthcare services, but do not automatically translate into adequate and acceptable services. Focussing attention on how services are delivered might restore the balance between supply (services) and demand (user needs) and promote universal and equitable access.

## Abstrait

**L'impact des variables des services de santé sur l'accès aux soins dans un cadre urbain à faibles revenus dans le Western Cape, Afrique du Sud.**

**Contexte:**

L'accès aux soins est complexe et polyvalent, et étant un droit fondamental, tous les groupes d'utilisateurs devraient avoir également accès à ses services.

**Objectifs:**

Le but de cet article est d'examiner l'impact de la prestation de services sur l'accès aux soins pour les groupes vulnérables dans un cadre urbain de soins primaires en Afrique du Sud.

**Méthodes:**

On a utilisé un modèle d'étude qualitative. On a recueilli les données au moyen d'entrevue semi-structurées avec des participants préalablement sélectionnés, puis on les a analysées au moyen d'une analyse thématique du contenu.

**Résultats:**

Les facteurs de prestation de services sont présentés par rapport à cinq aspects d'accès selon le Cadre ACCESS. Du point de vue du fournisseur, l'organisation des soins dans le cadre de l'étude a eu pour résultat des services disponibles, accessibles, raisonnables et adéquats par rapport aux politiques et directives du Système de santé de district. Cependant, les prestataires de service se sont heurtés à des obstacles considérables dans la prestation de services qui ont eu un effet sur la qualité des soins, et ont mal répondu aux besoins des clients et des prestataires, et ont fini par compromettre l'admissibilité des prestations de service. Bien que les utilisateurs aient trouvé que les services étaient accessible, l'organisation des services présentait des défis dans les domaines de la disponibilité, de la rentabilité et du caractère adéquat, ce qui a eu pour effet des besoins non satisfaits, peu de satisfaction et une perte de confiance. Ces défis ont nourri des sentiments de rejet des services.

**Conclusion:**

Les systèmes et l'organisation de services bien développés peuvent créer des services sanitaires primaires accessibles, abordables et disponibles, mais ne se traduisent pas automatiquement en services adéquats et acceptables. L'attention accordée à la façon dont sont fournis les services pourrait rétablir l'équilibre entre la fourniture (services) et la demande (besoins de l'utilisateur) et promouvoir un accès universel et équitable.

## Introduction

Access to healthcare is a basic human right,^1^ and governments should aim to provide universal and equitable access to high quality health care services.^2^ A feature of vulnerable populations may be the risk of less health care access and poorer health care outcomes than the general population.^3^ An exploration of the experiences of vulnerable groups can provide information on their access to, and satisfaction with, health care services.

A number of authors have tried to capture the complexity and multi-faceted nature of health care access through different frameworks.^2,4,5,6,7,8^ The more comprehensive of these frameworks, such as the Health Access Livelihood Framework (ACCESS) described below, acknowledges a dynamic interaction between demand (user) and supply (service).^2,5,6,8,9,10^ For instance, an accessible service will attempt to structure hours of operation (supply) in accordance with the schedule of users (demand).

In this article, ACCESS^2,9,10^ is used to explore health care access for vulnerable groups in a specific setting. According to this framework, healthcare access constitutes five dimensions: availability, accessibility, affordability, adequacy and acceptability, as defined in Table 1. These are influenced by a dynamic interaction with user livelihood assets (the human, social, physical, financial and natural assets, or capital, a person has access to)^11^ on the one hand, and policies, institutions, organisation and processes on the other.^2,6,8,9,10^

**TABLE 1:** The dimensions of access to healthcare services according to the ACCESS Framework.^2^

Dimension	Defintion^2p1586^	Aspects to consider^22^
Availability	‘The existing health services and goods meet clients` needs.’	Adequate supply of services, goods and facilities, including types of services, sufficient skilled human resources
Accessibility	‘The location of supply is in line with the location of clients.’	Proximity, means of transportation and travel time
Affordability	‘The prices of services fit the clients’ income and ability to pay.’	Direct and indirect costs of accessing health care
Adequacy	‘The organization of health care meets the clients’ expectations.’	Organisation of services, including the standard of the facilities and meeting user expectations
Acceptability	‘The characteristics of providers match with those of the clients.’	Ethical standards and the appropriateness of services, goods and facilities to address cultural and gender differences and life-cycle requirements; to improve outcomes; and to ensure confidentiality, effective communication and facilitating attitudes

As set out by Flaskeru and Winslwow^12^ (p. 70), ‘vulnerable populations are defined as social groups who have an increased relative risk (of-) or susceptibility to adverse health outcomes’. Typically, these include poor people and groups who experience stigma, discrimination and intolerance, and/or political marginalisation,^12^ and those whose human rights are violated. Understanding the experiences of vulnerable groups in relation to the five components of ACCESS can assist in changing the provision of health care to enhance health outcomes.

The focus of this article is on the impact of these five dimensions on the health care access of a group of vulnerable users, including people living in poverty, people living with HIV and/or AIDS or chronic conditions such as diabetes mellitus, people with disabilities (PWD), women, all members of women-headed households, youths, elderly people, members of minority cultures and persons with low levels of education and literacy.

## Research methods and design

### Study design

This article is based on the results from a large international study entitled ‘Enabling universal and equitable access to healthcare for vulnerable people in resource poor settings in Africa’.^13^ This article presents the results of one component of that study: the qualitative phase from one of the four South African (SA) sites. The larger study had three phases and was conducted concurrently in four countries in Africa and four sites in each country.^13,14,15^ The site for the study component reported in this article, an urban township in Cape Town, was purposively selected because it is a small, densely populated and impoverished urban community.

### Setting

Gugulethu, a small township (less than 10 km^2^) in the Klipfontein subdistrict of the Klipfontein and Mitchells Plain substructure of the City of Cape Town Metro health district, has a population density of 15 161.7 persons per km^2,16^ The population comprises mainly black Africans (98.58%)^16^ with only 3.6% being 65 years or older.^17^ Whilst 2.2% of the population is illiterate, 60% have not completed high school.^17^ Almost 40% of this poverty-stricken community was unemployed^16^ and 71.4% of households (average size of 3.33) had an income of R3200.00 (approx. $300.00) or less per month in 2011.^17^

### Study population and sampling strategy

The study population comprised the community and service providers in the public, traditional and private health care services in Gugulethu. Purposive and snowball sampling techniques were employed to identify a participant group with a wide diversity of experience and views.^18^

Eight public health care users, and four persons who stopped using public health care (non-users) were selected from various vulnerable groups in consultation with community leaders and non-governmental organisations. Table 2 provides an overview of the vulnerability profile of these participants. The numbers in the table total more than 12 as some participants fall into more than one group. The identification of vulnerable groups is described and defined by Mannan, Amin, MacLachlan and the EquitAble Consortium (2014)^19^ and Eide, Amin, MacLachlan, Mannan and Schneider (2013).^20^

**TABLE 2:** Vulnerability profile of health care users and non-users.

Vulnerability factor present	Non-users	Users
Poverty	4	8
HIV and AIDS	0	3
Chronic conditions, excluding HIV and AIDS	2	4
Physical disability	3	5
Women	3	4
Women-headed household	1	3
Aged	1	0
Youth	0	1
Minority groups (subculture)	0	1
Low level of education (Illiterate or education level of less than grade 7)	1	2

Health care providers were purposively sampled from the Community Health Centre (CHC) and two clinics. The first author used her knowledge of the health care system to identify the clinics, one east and the other west of the more centrally located CHC. One clinic was near an informal housing area, whilst the other in a slightly more resourced area with formal housing close to a shopping centre. The public healthcare providers interviewed included professional service providers, community health care workers, support staff (e.g. administrative, cleaning, security) and a community liaison officer. Table 3 sets out the occupational profile of the service providers at the public primary health care (PHC) facilities. One general practitioner and 12 traditional healers were also interviewed. However, as this article focuses on the public health care sector, these interviews were not included in the results presented in this article.

**TABLE 3:** Occupational profile of providers interviewed at the Primary Health Care facilities.

Post/category of provider	Number
Security staff	1
Cleaning staff	1
HIV and/or AIDS Counsellor	1
Health promoter	1
Administrative clerk	2
Enrolled nurse	2
Orthopaedic aftercare professional nurse	1
Community liaison officer	1
Clinical nurse care practitioner	1
Professional nurse	3
Physiotherapist	1
Family physician	1
Operation manager (professional nurse)	1
Orthotist-prosthetist	1
Social worker	1

### Data collection

Data were collected through 44 semi-structured interviews between April and November, 2010. Fourteen interviews (private general practitioner and traditional healers) were excluded in the results presented in this article since the data did not add any insights to the PHC service delivery process, which is the focus of this study. As an adjunct to the interviews, direct observation^18^ was done at the facilities, and institutional process and policy documents were perused by the primary author. Data on services provided, equipment, access and resources were captured on specifically designed data capture sheets.

The interviews were divided between research team members according to language proficiency and were conducted by the primary author (14 interviews), a co-researcher (2 interviews) and three research assistants (28 interviews). This might have added bias, since the primary author and co-researcher, unlike the research assistants, are graduates with experience in the fields of research and health care service delivery. The team was trained by the core research group. Additional training sessions for the research assistants were held by the primary author.

Interviews were conducted at venues determined by the participants and included the participants’ homes, places of work and public places such as community centres. The study was explained to each participant, and informed consent and permission to digitally record the interviews was obtained. Interviews lasted between 45 and 90 minutes.

### Data analysis

#### Data management and analysis

All interviews were transcribed verbatim and the isiXhosa and Afrikaans interviews translated into English. The one Afrikaans translation was verified by the primary author, an Afrikaans first language speaker; the isiXhosa translations were verified by an independent person employed in the public health care service, whose duties included interpreting, and who was not part of the EquitAble research team. Data were analysed using thematic content analysis to identify emerging and recurring themes. Individual experiences and factors relating to health care access were identified and coded from each transcript. Codes were then grouped together into themes. Some themes were predetermined based on the interview schedule, whilst new themes were identified as experiences were explored. Recurring themes were grouped together to eventually form three main themes, as set out in the results section.

#### Verification

Data coding was developed and verified, through discussion in the research team, to allow for comparative coding for the four SA sites.

Triangulation of data was done by comparing experiences of participant groups with one another and was augmented by direct observations and perusal of policy and procedure documents. Presentation of the results to the wider community provided a further opportunity to verify and triangulate the findings.

## Ethical considerations

The study was registered and approved by the Committee for Human Research of Stellenbosch University (reference number N09/10/270), and permission to perform the study was granted by the Western Cape Department of Health for the CHC and the City of Cape Town for the two clinics.

Identifiable information was depersonalised by means of coding along the guidelines developed for the EquitAble framework. All digital data were stored and backed up electronically using password protected entry to both the folders and files. Paper records were archived with Stellenbosch University.

## Results

Three main themes, namely service factors, personal factors and environmental considerations were identified. This article presents the findings on the service factors according to the five dimensions of access as defined by the ACCESS Framework:

- availability
- accessibility
- affordability
- adequacy
- acceptability.

Quotes from the interviews are provided as examples of common themes.

## Availability

Availability refers to the type of services offered, whether human and other resources are sufficient to meet the demand, and to the knowledge and skills of service providers.

### Type of services

Public healthcare services in Gugulethu are provided by a CHC and four clinics. Table 4 presents information on the services offered, staff complement and service hours of these facilities.

**TABLE 4:** Summary of the public health care services in Gugulethu.

Summary	Community health Centre (*n* = 1)	Clinics (*n* = 4)
Hours	7.30 am until 4.30 pm weekdays	7.30 am until 4.30 pm weekdays
	24 hours emergency and maternity services	
	Extended hours (Saturday and after hours clinics) for services such as women's health clinics, children and baby clinics to accommodate people who work.	
Health care staff	Medical officer	Professional nurse
	Family physician	Clinical nurse care practitioners
	Professional nurses	Other levels of nursing staff
	Clinical nurse care practitioners	Weekly consultant visits by clinical specialists
	Other levels of nursing staff	Community health workers
	Social workers	Counsellors
	Physiotherapists	Health care promoters
	Community health workers	
	Counsellors	
	Health care promoters	
	Outreach visits by the sub district occupational therapist and prosthetist from the provincial orthotic and prosthetic workshop	
Services offered	Full primary health care package	Health promotion
		Various preventative and curative care services

Although the services offered at the CHC and clinics are in line with District Health System (DHS) requirements (as noted in the process and policy documents perused), users expressed dissatisfaction as services were not always what they expected. For instance, the CHC had both emergency and rehabilitation services, but the clinics did not (see Table 4), a situation that users found unsatisfactory:

‘And they [*clinic*] don't have an emergency [*section*] – no matter how serious your situation.’ (Non-user, female, 35–49 years, single parent)

‘It's not right because I must see the physiotherapist every now and then. I sometimes don't have money but I'm forced to get it [*privately*] because there's nothing that I can get from clinic. Their service is very poor for me and if the condition doesn't change some people will always ignore to go there.’ (Non-user, female, 50–64 years, arthritis)

Observation showed that rehabilitation services were offered at the CHC only and was facility-based with a focus on treating acute conditions through individual sessions weekly, twice monthly, or monthly. Frequency of sessions is often determined by time constraints rather than the norm for specific conditions.

### Human resources

Providers complained that, although they had full staff complements in accordance with the approved post lists (APL), they were not sufficient for the number of patients. Service delivery was further compromised by absenteeism, leave and compulsory training:

‘We do have a high absenteeism rate – that's part of the problem. So you've got … 15 nurses on the staff but there's only seven of them on duty because two of them are on a course, two are on leave and three are off sick, you know. So actually on paper our staff is enough, but maybe on the floor we don't have enough because of absenteeism.’ (Healthcare professional, female, 34–49 years)

Rehabilitation services were provided by one specialised professional nurse and one physiotherapist who also served the larger district. The physiotherapist was based at the CHC whilst the professional nurse was based outside the study area:

‘… sometimes I only get to see a patient every two weeks which is not ideal but that is the best that I can come up with … some patients they require it [*treatment*] immediately and I'll try and see to those patients, but otherwise it is really difficult … And I would try and give them an exercise program to follow in that time …’ (Healthcare professional, female, < 34 years)

### Provider's knowledge

Providers felt that they lacked the necessary knowledge and skills to manage health care for PWD and that the support systems were inadequate:

‘We're just loaded with everything. I would say that the Department of Health is just loading us because it wants us to do everything yet we don't have the skills or the facilities to refer to.’ (Health care professional, female, 50–64 years)

Users with disabilities concurred with this view:

‘They [*the staff*] do not have … the right understanding … our disabilities are different, therefore, also the approach is supposed to be different.’ (User, male, 35–49 years, ankylosing spondylitis)

‘I thought if they communicate with people or patients, for instance in my case they should have approached my special doctor [*clinical consultant at tertiary hospital*] for advice … but they did not listen to me.’ (User, male, 35-49 years, ankylosing spondylitis)

Users in need of comprehensive rehabilitation or medical management of impairments underlying their disabilities are referred to services outside of the area but service providers did not appear well informed of these referral pathways:

‘I think it is more difficult now for the people to get what they need, because there are just so few people who really know what is going on. It takes a long time before the patients are being directed in the proper channels.’ (Healthcare professional, disability-specific services, female, 34–49)

According to the providers many referral services had backlogs, with waiting periods as long as two years.

### Equipment, resources and assistive devices

Periodic shortages of equipment and resources were experienced, particularly in the trauma unit of the CHC:

‘This month it could be gloves. Next month it could be oxygen masks …’ (Health care professional, female, 50–64 years)

In addition, administrative delays were reported with the purchasing of consumables and the replacement of condemned equipment. Equipment was reportedly shared between departments and consultation rooms in the CHC and clinics, leading to time wasted searching for it:

‘… your practitioners are spending most of their time running around to borrow this or to find that. That is not a system that can work, you know.’ (Health care professional, female, 50–64 years)

Whilst some providers found creative solutions, others were unable to deliver services without all available equipment:

‘I mean, we sometimes run out of a certain size of bandages; I just saw [*name removed*] cutting a bandage in half for cost-effectiveness. So some people will be creative because we really do have the patients’ needs as a priority.’ (Health care professional, female, 50–64 years)

There was an ample supply of assistive devices, such as basic folding frame wheelchairs, walking sticks and crutches at the CHC. However, periodic shortages of consumables like catheters and stoma bags were reported, and although users are notified and supplied immediately when consumables are again available, they may be without these necessary health supplies for short periods.

In summary, providers and users agreed that service availability was challenged by a lack of equipment and consumables and too few service providers. Services to PWD specifically were further hampered by a lack of disability-specific knowledge, not enough human resources and the way in which services were delivered.

## Accessibility

In the context of accessibility within the ACCESS Framework,^2^ participants were positive about the proximity of the health care facilities to their homes. They lived within a 3 km radius of the services and most accessed the facilities on foot. The accessibility of the facilities was overall good for PWD.

## Affordability

Affordability refers to the direct costs of care as well as indirect costs such as travel costs, lost time and loss of income.^2^ All PHC services were delivered free of charge.

According to the liaison officer, long waiting times was the most common complaint received from users. Waiting times varied between two hours for those with scheduled appointments and four hours or longer for those without appointments.

Users employed strategies to decrease waiting times such as asking staff or other users to take their registration cards to the facility ahead of their arrival:

‘Some of them in the neighbourhood ask us, which makes things difficult for us. An old person knocking at your door in the morning – “please can you take my card in there”. You can't say no to an older person …’ (Health care services staff, non-professional services, female, 50–64 years)

## Adequacy

### Organisation of services

In accordance with DHS guidelines users had to access the facility (CHC or clinic) that provided services to the geographic service area (GSA) in which they live. However, some users preferred to access the CHC instead of the clinic in their GSA. They accessed the CHC after normal clinic hours knowing that, in accordance with policy, they would not be turned away:

‘I think they're [*the patients*] running away from their clinics … So they will wait until their clinic is closed and then definitely we must admit everybody …’ (Health care professional, female, 50–64 years)

In addition, according to set referral pathways, users are not allowed to access secondary or tertiary level services without a referral from primary level:

‘I don't have a problem to go to hospital. But the road to hospital is via the clinic … I can't go there.’ (Non-user, female, 35–49 years, single parent)

As reported by providers and documents reviewed, in excess of 1500 patients are seen daily at the CHC and at the two clinics investigated. Systems such as appointments and 6-monthly prescriptions for chronic medications were introduced to reduce overcrowding, improve patient management and flow and to contribute to patient-focussed care. Extended service hours and outreach services further improve both access and patient flow. Triage systems at all entry points screen and prioritise unscheduled users. The elderly and PWD receive preferential services. Service delivery is divided into dedicated service streams such as diabetes, hypertension, psychiatric and HIV clinics. These clinics are open on specific days and at specific times (see Table 4). This can create access barriers as, for instance, early morning appointments may be difficult for those with disabilities:

‘But I don't always make it (to appointments) when they give me the time and maybe the afternoon can be better for me to attend.’ (User, female, 35–49 years, wheelchair user and partially sighted)

‘In my case, a disability person, I send someone in the morning to stand [*in the queue*] for me … Due to the fact that I am crippled it takes too long for me to reach the clinic early.’ (User, female, 35–49 years, post-polio, HIV and/or AIDS, psychiatric condition)

In the past, users were seen on a first come, first serve basis, which resulted in long waiting times and people queuing hours before opening time, often in the dark, with concomitant safety risks of traveling from home in the dark. Despite significant positive changes to improve patient flow, negative perceptions continued to dominate the decision of non-users:

‘No, I stopped (going to the clinic). I almost got killed … one of them had a gun against me …’ (Non-user, female, 80 + years, arthritis)

‘… I can't go to clinic – wake up by 4 am while I'm sick – it's a huge risk to my life.’ (Non-user, female, 35–49 years, single parent)

Similarly, the past lack of systematic management of the patient load causing backlogs and frustration still continues to influence decisions of non-users to not use PHC services:

‘I was the first one in and put my medical card on the nurse's desk but as other people came in, their cards piled up on top of mine and I ended up being the last one. I got so upset and ended up slapping one of the nurses.’ (Non-user, female, 80 + years, arthritis)

Users cannot request a specific health care provider, and follow-up appointments are not made with the same provider. In addition, students and community service providers rotate through the services, often on a monthly basis. These practices impacted negatively on continuity of care and led to poor follow up:

‘Last year I went to the clinic to collect the test results of my father with a letter that shows to them what was done, but each and every one sent me to someone else … They show the signs of lack of understanding and incompetence.’ (User, female, 20–34 years, post-polio, HIV and/or AIDS, single parent)

### Meeting user expectations

The second most common complaint the community liaison officer at the CHC dealt with was unmet user expectations:

‘… they complain that the doctor did not treat them according to their specifications.’ (Health care support staff, male, 34–49)

‘I was the one expecting them to take an X-ray for my chest pain but they never did that.’ (User, female, 20–34, post-polio, HIV and/or AIDS, single parent)

‘At [*name of tertiary hospital*] I was given a letter to give to [*name removed*] clinic for my treatment. When I go there they give me totally different medication. I do not know if that medication is going to help …’ (User, female, 35–49 years, post-polio, HIV and/or AIDS, psychiatric condition)

‘Like sometimes they [*nursing staff*] write you a prescription and then you ask to see a doctor, then they will shout at you and ask why … They are not doctors … In my case I told them I'm the one who is sick here. I want to see a doctor or I will phone Manta, [*previous*] Minister of Health and tell her that you do your [*swear word*] here.’ (User, female, 35–49 years, post-polio, HIV and/or AIDS, psychiatric condition)

In summary, despite efforts to improve adequacy, defined as the organisation of care, and the extent to which services met the expectations of users, such as the introduction of GSA referral systems, the number of clinics and diversity of services provided, extended hours, outreach services, triage systems, preferential treatment for the elderly and PWD, six monthly prescriptions and organising the services into disease specific clinics, users felt that services did not adequately meet their needs.

## Acceptability

### Attitudes

Some users found providers to be caring, positive, committed and professional and felt that they were treated in an acceptable manner:

‘Some of the staff are very organised and committed to serve people. They treat us equally and they keep your matters confidentially. So far I'm still satisfied about the way they treated me.’ (User, male, 20–34 years, diagnosis unknown)

‘I use the clinic because they give my treatment and explain to me the direction to use and if I cheat [*on*] my medication they also tell me what is going to happen …’ (User, male, 20–34 years, epilepsy)

Other users experienced the services as unacceptable. They felt providers were disrespectful, rude, uncaring and rushed:

‘… the nurses treat them with no respect.’ (User, female, 20–34 years, post-polio, HIV and/or AIDS, single parent)

‘And you end up sacrificing your last money to go to the private doctor to avoid humiliation because of the behaviour of the staff.’ (User, male, 35–49, amputee)

‘When they [*the nurses*] give directions [*about taking medication*] they talk so fast. As a result you get lost when you are at home. You ended up taking a wrong medication because that person never checked your understanding by that time because she is in a hurry.’ (User, female, 20–34 years, post-polio, HIV and/or AIDS, single parent)

‘… when you tell the doctor what you have like headache, swollen feet and thrush, the doctor response will say, ‘Do not mention everything! You did not come here to do some grocery shopping.’ (User, female, single parent, 15–19 years, HIV and/or AIDS)

Providers had been accused of favouritism and nepotism:

‘… there is favouritism – they treat better their families and friends. When their friends come, they give them folders before us and they finish sooner than those of us who were there from early in the morning.’ (User, female, 20–34 years, post-polio, HIV and/or AIDS, single parent)

When asked about these user comments, providers acknowledged negative attitudes:

‘It is difficult. Let me see. That's a tricky one because in any environment you've got good potatoes and rotten ones so the truth is that you‘ll find those that will really not work well with the patients, you see. But obviously from time to time I will reprimand them you see? Yes, [*the complaints are usually*] from the same person or the same department. There are those cases – it is an open secret that we cannot hide.’ (Health care support staff, male, 34–49 years)

Stringent confidentiality policies and practices inadvertently place the health care support staff in a dilemma and portray them as unhelpful or uncaring:

‘… at times a patient might come out the doctor's consulting room, they will come to you – ‘Where must I go now’? The doctor has told the patient to go to a certain place, but as soon as they come out of this door, the first person they meet they ask, ‘Where must I go to now’. And I as a worker here, I do not have the right to open that folder to guide me where is this person supposed to go. I do not have that right. For that person it must be strange for not knowing where must they go, so my answer to that person will be, ‘Go back to the doctor and ask him where you must go to.’ (Health care services staff, non-professional services, female, 50–64 years)

Yet, at the same time, confidentiality is unintentionally breached by the organisation of services:

‘There is no confidentiality because if you are HIV and/or AIDS or diabetic there are different sides for those diseases. I felt that is wrong because if diagnosed with HIV most of the time you are not ready to be known by other people. They embarrass us because they will call loudly saying: ‘Those who came for antiretroviral drugs that side and the result of HIV that side.’ (User, female, single parent, 15–19 years, HIV and/or AIDS)

User behaviour and low morale amongst providers contributes to negative attitudes:

‘So even patients themselves … they can be very bad. You see? Sometimes some of them come drunk … But sometimes you try to understand their problems because this person is hungry, he is coming from poverty he is vulnerable and he is sick … yes, and that person will take it out on the staff. And they even do it to me sometimes … These behaviours are normally seen over weekends and after four … They come smoking, drinking and all those problems … Or they come during gang fights … some of the gangs will be bringing in their friend they will demand that everything stops … that this is the patient that needs to be seen … Yes, that person must be prioritised and others will just drink and shout and swear, you see?’ (Health care support staff, male, 34–49 years)

‘You will find that in terms of caring for them [*providers*] and supporting them and acknowledging the hostile environment that they are working in and the situation and the long hours that we work there isn't much of an appreciation. So the morale is not that high. Sometimes they will complain that when they work overtime it will take 4 to 5 months for their overtime to be paid.’ (Health care support staff, male, 34–49 years, tertiary education)

Furthermore, assumptions and stereotyping exclude PWD from general healthcare practices and access:

‘Even the HIV and/or AIDS diseases nurse will say: ‘Hee- hee! Where did you get it?’ It does not register to them that you are sexual active and you have blood. Even if you … are pregnant they will asked why are you pregnant, how this person make you pregnant … by saying how many children do you have and when you tell them they make a joke of you and the other patients will laugh at you and you became frustrated and angry, all of that.’ (User, female, 20–34 years, post-polio, HIV and/or AIDS, single parent)

Similar examples of stigma and discrimination included not giving pamphlets on sexually transmitted infections (STI) to a physically disabled person but to others, giving a room number to a partially sighted patient to find unassisted, and disability accessible toilets being used as storage rooms and kept locked.

### Language

Language barriers existed. The majority of the nursing, administrative and support staff spoke isiXhosa, compared to only one of the rest of the professional staff and interns. Although users did not report language to be a barrier, providers frequently did. Since there are no formally trained interpreters, bilingual staff act as interpreters and inadvertently increase their own and other staff-members’ workloads:

‘Sometimes the issue of the language. Maybe the doctor … they [*the doctor and the user*] did not understand each other properly, you see? … but we did try and address it and said there must be an assistant nurse to interpret for the doctor, but sometimes you don't have enough staff to do such things. There are other core businesses and a nurse will sit there and just interpret for the doctor …’ (Health care support staff, male, 34–49 years)

In general, many users experienced barriers with regards to the acceptability of services, particularly in the form of provider attitudes and the impact of diagnoses-based organisation of services on confidentiality and poor communication. Acceptability of service delivery was compromised for service providers through negative user behaviour, language as barriers, short contact sessions and fragmentation of services.

## Discussion

Considering the historical context of a fragmented and inefficient healthcare system with poor capacity,^8^ remarkable achievements in health care service delivery were observed in the PHC health care facilities of Gugulethu. From a service provider's perspective the results demonstrate available, accessible, affordable and adequate services in the study setting through efficient organisation of services according to the DHS guidelines and policies. However, user perspectives differed. Although services were accessible, challenges were experienced with regard to affordability, availability and adequacy. The discussion will show how challenges in health care service delivery created conflict between users and providers, contributed to unacceptable behaviour from both groups, eroded trust, and led to decreased satisfaction and quality of care and, ultimately, resulted in unacceptable services for both groups.

Despite full staff complements according to DHS guidelines, daily availability was compromised by a lack of human resources, with providers seemingly under pressure. Care was punctuated by rushed consultations, long waiting times, fragmentation and poor continuity, which together with limited time for patient education culminated in errors and perceptions of poor quality care and a lack of satisfaction amongst users and providers.

Long waiting times seem to be characteristic of the SA healthcare system.^4,14,21,22^ Strategies by users to decrease waiting times may put providers in a difficult position. Refusing assistance was perceived as uncaring and assisting their actions was perceived as favouritism. These perceptions, in conjunction with impatience and frustration at long waiting times, may negatively influence user attitudes and lead to impatience and rudeness.

PHC services are largely nurse-driven, but for many users this impacts on adequacy and acceptability of services. Previous studies on nurse-driven services^23,24^ found high patient satisfaction rates as nurses spent more time and provided more information and counselling than doctors. Unfortunately the demand on the services might have prevented a similar finding in this study setting. In addition, it seems that, for some users, the traditional picture of the doctor as the PHC provider was strongly embedded and thus a key expectation, as described by Branson et al.^24^ Users might view treatment by a nurse as a compromise in quality of care^25^ especially in an urban context where expectations of care from a medical doctor are high and not unreasonable.

Large numbers of patients are effectively managed and waiting times reduced through organising services into diagnostic clinics such as HIV, diabetes, hypertension and arthritis clinics. However, such organisation impacted negatively on user privacy and confidentiality and the acceptability of the services. Merely attending a specific clinic or unit robbed the user of confidentiality as their health status was publically displayed. Such unintentional breaches in confidentiality may act as powerful deterrents to accessing public health care services. In addition, this constitutes an impairment-oriented approach that depersonalises the user,^26,27^ compromises holistic, patient-centred care,^27,28^ as well as continuity and coordination of care.^28^ Whilst promoting standardised care and protecting providers from full personal contact with users, it also limits provider work satisfaction and fuels stress and anxiety.^27^

Acceptability of the services was limited as users were disempowered through lack of choice, thus affecting quality of care and satisfaction.^28^ They could not choose which facility to use, the service provider they would like see, nor the day or time of their appointment. There was no trust relationship to explore user expectations.

Unmet expectations fuelled perceptions of inadequacy and unacceptability. According to Dixon-Woods and colleagues,^7^ unmet needs exist as a result of the conflict between health services seeking to constitute and define the appropriate objects of medical care versus what the user defines as the focus of care. The outcome of this continuous reinforcing of conflict dynamically shapes access^7^ and perceptions of the quality of care.^28,29^ For example, the user expectation of the availability of an emergency service at the clinics may not be that of health providers. Setlhare and colleagues^30^ emphasised the need for context-specific patient-provider models which are sensitive to cultural and regional constructs. In the current study, some users asserted themselves by accessing the CHC after hours. However, such a strategy can impact negatively on planning, services and resources.^31^ In addition, providers felt taken advantage of, which may have resulted in negative attitudes towards users.

Past studies^4,32,33,34,35^ have demonstrated how working conditions lead to poor staff morale and negative attitudes and affect user satisfaction and quality of care. Staff morale in this study was eroded by inadequate numbers of service providers, high turnover of providers and interns, periodic shortages of equipment and/or consumables, language barriers and the absence of interpreters, time pressures, working in an unsafe community and with users who may be rude, abusive and violent. Together these factors may result in already tenuous interpersonal relations culminating in negative attitudes and behaviours.

Overall the behaviour of both providers and users in this study demonstrated little mutual respect, empathy and tolerance. Unfortunately negative attitudes and unprofessional behaviour of providers noted in this study have all too often been documented within the SA health care literature.^4,21,22,31,36,37,38^ Although limited to certain individuals, these attitudinal barriers may well end the user's relationship with the facility,^4,37,39^ as was evident in this study. The impatient, rude and abusive behaviour of users may demonstrate their lack of trust in and respect for the provider-patient relationship. On the other hand, such behaviours result in providers feeling negative towards users. Expanding the human resource component of service provision will improve capacity of services and might result in an improvement in attitudinal challenges, as demonstrated in previous research.^31^ Although many strategies were already implemented to improve efficiency, flow management and referral procedures, potential to improve leadership and the quality of management within facilities should also be explored.

Ongoing trust relationships form the foundation of client centred healthcare^39^ and are especially important in the management of chronic diseases.^28^ PWDs often suffer from chronic conditions or require ongoing healthcare due to the nature of their impairments,^40,41^ but are twice as likely than their non-disabled peers to experience inadequate care at health facilities.^42,43,44,45,46^ PWDs in this study were no exception with both healthcare providers and users alluding to this fact. Their healthcare was compromised by a lack of rehabilitation service providers, stereotyping and a lack of skills and knowledge. They often faced delayed referral and long waiting times to access these referral services. Waiting times can lead to a deterioration of the health condition and impairment and can aggravate the disability or turn a temporary disability into a permanent one.^41^ Stereotyping, from lack of knowledge, caused discrimination, rudeness and exclusion from important services. The need for more training and support for primary care providers in the comprehensive management of chronic and more complex conditions has been recognised before.^14,47^ In the climate of re-engineering of PHC^48,49,50^ and provision of a continuum of all four dimensions of care at primary level (promotive, preventive, curative and rehabilitation services), the poor availability of rehabilitation-specific services on the PHC platform in Gugulethu was concerning.

In summary, providers experience significant stressors in their efforts at providing satisfactory heath care, despite many positive features and favourable impressions of the services reported by users. These were, however, quickly overshadowed by negative experiences and perceptions, leaving users feeling disempowered and voiceless, victimised and betrayed by the very system that is supposed to enhance their well-being. Their desperation can be summarised by the lament of this user who responded as follows when her health care needs were not met:

‘My heart is becoming broken.’ (User, female, single parent, 15–19, HIV and/or AIDS)

## Limitations

The qualitative nature of this study limits the generalisability of the results to a wider context.

The language and cultural barrier between the primary researcher and the participants may have affected the depth of experiences explored, especially where an interpreter was used or both parties conversed in their second language.

## Implication and recommendations

Considering the limitations of this study and the multi-dimensional facets of health care access, recommendations pertain to the study site only. Some of the recommendations may be applied in other settings after careful consideration of contextual differences and similarities.^51^

Whilst the recommendations are based on the service dimension only, the authors second the need for research in the development of context-specific patient-provider models^30^ where the emphasis is on user constructs. These should include holistic health care provider models with a focus on personal continuity and choice, as well as the management and inclusion of PWD and disability-specific services.

In particular, the lack of communication about the service structure and function seems to be an important factor which perpetuates negative perceptions of the services. Community information strategies^52^ must therefore be employed to inform and educate^24^ the community on the scope and structure of health care services and to market positive changes in services.

Codes of conduct for users (Patient Rights Charter)^53^ and providers (the Public Servants Code of Conduct^54^ and the principles of the Batho Pele [People first] initiative^55^) are systems measures aimed at achieving service acceptability. However, the failure of these strategies to effect change indicates the need for innovative strategies which consider both the user and the service.

## Conclusion

The study showed that efficient administrative and logistical organisation of health care service and systems does not automatically translate into adequate and acceptable services from a user's perspective. The balance can be restored by changing how services are delivered and how users are informed. Service delivery should include a patient-centred approach with consideration of aspects such as choice, comprehensive individualised care, continuity of care, shared consultation and participative decision making, non-discrimination, as well as good communication with a focus on mutual respect and courtesy.

Restoring the balance between service provision and user demands should facilitate universal access and equitable health care service delivery, particularly for vulnerable groups, and ensure that the public PHC services become the key to the management of health, as was stated by one of the participants:

‘The clinic is a very most important place to be because it is the key to any health centre or doctor.’ (Non-user, female, 35-49, single parent)

## References

[1] World Health Organization Human rights, health and poverty reduction strategies. Geneva: WHO; 2008.

[2] Obrist B, Iteba N, Lengeler C, et al. Access to healthcare in contexts of livelihood insecurity: A framework for analysis and action. PLOS Med. 2007;10(4):1584–1588. 17958467 10.1371/journal.pmed.0040308 PMC2039761

[3] World Health Organization Poverty reduction strategy papers Their significance for health: second synthesis report. WHO: Geneva, Switzerland; 2004.

[4] Harris B, Eyles J, et al. Adverse or acceptable: Negotiating access to a post-apartheid healthcare contract. Globalization and Health [serial online]. 2014 [cited 2014 June 13];10:35 Available from: http://www.globalizationandhealth.com/content/10/1/35. 10.1186/1744-8603-10-35 PMC4036079 24885882

[5] Levesque JF, Harris MF, Russel G. Patient-centred access to health care: Conceptualising access at the interface of health systems and populations. Int J Equity Health [serial online]. 2013;12:18 Available from: http://www.equityhealthj.com/content/12/1/18. http://dx.doi.org/10.1186/1475-9276-12-18 10.1186/1475-9276-12-18 PMC3610159 23496984

[6] Gilson L, Schneider H. Understanding health service access: Concepts and experience. Global Forum Update Research Health. 2007;4:28–32.

[7] Dixon-Woods M, Cavers D, Agarwal S, et al. Conducting a critical interpretive synthesis of the literature on access to healthcare by vulnerable groups [serial online]. 2006 [cited 2014 June 13];6:35. doi:10.1186/1471-2288-6-35 Available from: http://www.biomedcentral.com/1471-2288/6/35. PMC1559637 16872487

[8] Oliver A, Mossialos E. Equity of access to healthcare: Outlining the foundations for action. J Epidemiol Community Health. 2004;58:655–658. http://dx.doi.org/10.1136/jech.2003.017731 15252067 10.1136/jech.2003.017731 PMC1732867

[9] Balen J, Liu Z-C, McManus DP, et al. Health access livelihood framework reveals potential barriers in the control of Schistosomiasis in the Dongting Lake area of Hunana Province, China. PLOS Negl Trop Dis [serial online]. 2013 [cited 2014 June 12];7(8):e2350. doi:10.1371/journal.pntd.0002350 PMC3731233 23936580

[10] Bakeera SK, Galea S, Pariyo GW, et al. Community perceptions and factors influencing utilization of health services in Uganda. Int J Equity Health [serial online]. 2009 [cited 2014 June 12];8:25 doi:10.11861475-9276-8-25 Available from: http://www.equityhealthj.com/content/8/1/25. 10.1186/1475-9276-8-25 PMC2717964 19602244

[11] Department for International Development Sustainable livelihoods guidance sheets: Chapter 2 Framework [homepage on the Internet]. No date [cited 2009 Oct 19]. Available: http://www.livelihoods.org/info/info_guidancesheets.html.

[12] Flaskerud JH, Winslow BJ. Conceptualizing vulnerable populations health-related research. Nurs Res. 1998;47(2):69–78. http://dx.doi.org/10.1097/00006199-199803000-00005 9536190 10.1097/00006199-199803000-00005

[13] EquitAble Enabling universal and equitable access to healthcare for vulnerable people in resource poor setting in Africa [homepage on the Internet]. No date [cited 2014 May 28]. Available from: http://www.equitableproject.org.

[14] Visagie S, Schneider M. Implementation of the principles of primary healthcare in a rural area of South Africa. Afr J Prm Healthcare Fam Med [serial online]. 2014 [cited 2014 May 29];6(1). Available from: http://dx.doi.org/10.4102/phcfm.v6i1.562 10.4102/phcfm.v6i1.562 PMC4502891 26245391

[15] Braathen SH, Vergunst R, Mji G, et al. Understanding the local context for the application of global mental health: A rural South African experience. Int Health [serial online]. 2013;5(1):38–42. doi:10.1093/inthealth/ihs016 24029844

[16] Frith A. Census 2012: Gugulethu [homepage on the Internet]. No date [cited 2014 May 29]. Available from: http://census2011.adrianfrith.com/place/199030.

[17] City of Cape Town 2011 census suburb Gugulethu [homepage on the Internet]. 7 2013 [cited 2014 May 29]. Available from: http://www.capetown.gov.za/en/stats/2011CensusSuburbs/2011_Census_CT_Suburb_Gugulethu_Profile.pdf.

[18] Domholt E. Rehabilitation research Principles and applications. 3rd ed Elsevier: Missouri; 2005.

[19] Mannan H, Amin M, MacLachlan M, EquitAble Consortium The EquiFrame manual: a tool for evaluating and promoting the inclusion of vulnerable groups and core concepts of human rights in health policy documents. 2nd ed Dublin: Global Health Press; 2014.

[20] Eide AH, Amin M, MacLachlan M, et al. Addressing equitable health of vulnerable groups in international health documents. ALTER, Eur J Disability Res [serial online]. 2013;7:153–162. doi:10.1016/j.alter.2013.04.004

[21] Hasumi T, Jacobsen KH. Healthcare service problems reported in a national survey of South Africans. Int J Qual Health Care [serial online]. 2014 [cited 2014 June 1]. Available from: http://intqhc.oxfordjournals.org/. 10.1093/intqhc/mzu056 24840002

[22] Day C, Gray A. Health and related indicators In: Barron P, Roma-Reardon J, editors South African Health Review 2008. Durban: Health Systems Trust; 2008 p. 239–396.

[23] Delamaire M, Lafortune G. Nurses in advanced roles: A description and evaluation of experiences in 12 developed countries. OECD Health Working Papers No 54, OECD Publishing [homepage on the Internet]. 2010 [cited 2014 June 12]. doi:10.1787/5kmbrcfms5g7-en http://dx.doi.org/10.1787/5kmbrcfms5g7-en

[24] Branson C, Badger B, Dobbs F. Patient satisfaction with skill mix in primary care: A review of the literature. Primary Healthcare Res Dev. 2003;4:329–339. http://dx.doi.org/10.1191/1463423603pc162oa

[25] Kapp R, Mash RJ. Perceptions of the role of the clinical nurse practitioner in the Cape Metropolitan doctor-driven community health centres. SA Fam Pract. 2004;46(10):21–25.http://dx.doi.org/10.1080/20786204.2004.10873150

[26] Mosadeghrad AM. Healthcare service quality: Towards a broad definition. Int J Health Care Qual Assur [serial online]. 2013 [cited 2014 June 12];26(3):203–219. doi:10.1108/09526861311311409 Available from: http://www.emeraldinsight.com/0952-6862.htm 23729125

[27] Van der Walt H, Swartz L. Isabel Mensies Lyth revisited. Institutional defences in public health nursing in South Africa during the 1990s. Psychodyn Couns. 1999;5:483–495. http://dx.doi.org/10.1080/13533339908404985

[28] Sofaer S, Firminger K. Patient perceptions of the quality of health services. Annu Rev Public Health. [serial online]. 2005 [cited 2014 June 9];26:513–559. http://dx.doi.org/10.1146/annurev.publhealth.25.050503.153958 10.1146/annurev.publhealth.25.050503.153958 15760300

[29] Bell R, Kravitz RL, Thom D, et al. Unmet expectations for care and the patient-physician relationship. J Gen Intern Med. 2002;17:817–824. http://dx.doi.org/10.1046/j.1525-1497.2002.10319.x 12406352 10.1046/j.1525-1497.2002.10319.x PMC1495125

[30] Setlhare V, Couper I, Wright A. Patient-centredness: Meaning and propriety in the Botswana, African and non-Western contexts. Afr J Prm Health Care Fam Med [serial online]. 2014 [cited 2014 June 22];6(1). doi:10.4102/phcfm.v6i1:554. PMC5337990 26468553

[31] Masango-Makgobela A, Govender I, Ndimande JV. Reasons patients leave their nearest healthcare service to attend Karen Park Clinic, Pretoria North. Afr J Prm Healthcare Fam Med [serial online]. 2013 [cited 2014 June 16];5(1). doi:10.4102/phcfm.v5i1.559.

[32] Tshitangano TG. Factors that contribute to public sector nurses’ turnover in Limpopo province of South Africa. Afr J Prm Health Care Fam Med [serial online]. 2013 [cited 2014 June 22] 5(1). doi:10.4102/phcfm.v5i1.479

[33] Gross K, Pfeiffer C, Obrist B. “Workhood” – a useful concept for the analysis of health workers'resources? An evaluation from Tanzania. BMC Health Serv Res [serial online]. 2012 [cited 2014 May 28];12:55 Available from: http://www.biomedcentral.com/1472-6963/12/55. 10.1186/1472-6963-12-55 PMC3330008 22401037

[34] Karliner LS, Jacobs EA, et al. Do professional interpreters improve clinical care for patients with limited English proficiency? A systematic review of the literature. Health Serv Res [serial online]. 2007 [cited 2014 June 16];42(2):727–754. doi:10.1111/j.1475-6773.2006.00629.x PMC1955368 17362215

[35] Newman K, Maylor U. Empirical evidence for “the nurse satisfaction, quality of care and patient satisfaction chain”. Int J Health Care Qual Assur [serial online]. 2002 [cited 2014 June 13];15(2):80–88. Available from: http://www.emeraldinsight.com/0952-6862.htm.

[36] Lotika AA, Mabuza LH, Okonta HI. Reasons given by hypertensive patients for concurrently using traditional and Western medicine at Natalspruit Hospital in the Gauteng Province, South Africa. Afr J Prm Health Care Fam Med [serial online]. 2013 [cited 2014 June 22];5(1). http://dx.doi.org/10.4102/phcfm.v5i1.458

[37] Schneider H, le Marcis F, Grard J, et al. Negotiating care: patient tactics at an urban South African hospital. J Health Serv Res Policy. 2010;15(3):137–142. http://dx.doi.org/10.1258/jhsrp.2010.008174 20360145 10.1258/jhsrp.2010.008174

[38] Jewkes R, Abrahams N, Mvo Z. Why do nurses abuse patients? Reflections from South African obstetric services. Soc Sci Med. 1998;47(11):1781–1795. http://dx.doi.org/10.1016/S0277-9536(98)00240-8 9877348 10.1016/s0277-9536(98)00240-8

[39] Logie DE, Rowson M, Jugisha NM, et al. Affordable primary health care in low-income countries: Can it be achieved? Afr J Prm Health Care Fam Medn [serial online]. 2010 [cited 2014 June 22];2(1). doi:10.4102/phcfm.v2i1.246 http://dx.doi.org/10.4236/health.2014.65058

[40] Park JM. Disability and health service utilization among old Koreans. Health [serial online]. 2014;6(5):404–409. doi:10.4236/health.2014.65058 http://dx.doi.org/10.4236/health.2014.65058

[41] Shakespeare T. Disability rights and wrongs revisited. 2nd ed London: Routledge; 2014.

[42] Story MF, Kailes JI, Mac Donald C. The ADA in action at healthcare facilities. Disabil Health J. 2010;3:245–252. http://dx.doi.org/10.1016/j.dhjo.2010.07.005 21122793 10.1016/j.dhjo.2010.07.005

[43] Yee S, Breslin ML. Achieving accessible healthcare for people with disabilities: Why the ADA is only part of the solution. Disabil Health J. 2010;3:253–261. http://dx.doi.org/10.1016/j.dhjo.2010.07.006 21122794 10.1016/j.dhjo.2010.07.006

[44] Story MF, Schwier E, Kailes JI. Perspective of patients with disabilities on the accessibility of medical equipment: Examination tables, imaging equipment, medical chairs, and weight scales. Disabil Health J. 2009;2:169–179. http://dx.doi.org/10.1016/j.dhjo.2009.05.003 21122757 10.1016/j.dhjo.2009.05.003

[45] Graham CL, Mann JR. Accessibility of primary care physician practice sites in South Carolina for people with disabilities. Disabil Health J. 2008;1:209–214. http://dx.doi.org/10.1016/j.dhjo.2008.06.001 21122731 10.1016/j.dhjo.2008.06.001

[46] Gulley SP, Altman BM. Disability in two healthcare systems: access, quality, satisfaction, and physician contacts among work-age Canadians and Americans with disabilities. Disabil Health J. 2008;1:196–208. http://dx.doi.org/10.1016/j.dhjo.2008.07.006 21122730 10.1016/j.dhjo.2008.07.006

[47] Mash B, Fairall L, Adejayan O, et al. A morbidity survey of South African primary care. PLOS ONE [serial online]. 2012 [cited 2014 June 19];7(3):e32358 http://dx.doi.org/10.1371/journal.pone.0032358 10.1371/journal.pone.0032358 PMC3306367 22442666

[48] Naledi T, Barron P, Schneider H. Primary Healthcare in SA since 1994 and implications of the new vision for PHC re-engineering. South African Health Review In: Padarath A, English R, editors South African Health Review 2011. Durban: Health Systems Trust, 2011; p. 17–28.

[49] Western Cape Government: Health. Annual performance plan 2012/13 Cape Town: Department of Health; 2012 Available from: https://www.westerncape.gov.za/text/2012/3/doh_app_20122013.pdf.

[50] Western Cape Government: Health 2020: The future of health care in the Western Cape: A draft framework for dialogue. Cape Town: Department of Health, South Africa; 2011 Available at: https://www.westerncape.gov.za/other/2011/12/healthcare_2020_-_9_december_2020.pdf. http://dx.doi.org/10.1002/casp.2124

[51] Hodgetts DJ, Stolte OEE Case-based research in community and social psychology: Introduction to the special issue. J Community Appl Soc Psychol. 2012;22(5): 379–389.

[52] Ensor T, Cooper S. Overcoming barriers to health service access: Influencing the demand side. Health Policy Plan [serial online]. 2006 [cited 2014 June 12];19(2):69–79. Available from: http://heapol.oxfordjournals.org. 10.1093/heapol/czh009 14982885

[53] South Africa. Department of Health Patient rights charter [homepage on the Internet]. 2007 [cited 2014 May 30]. Available from: http://222.doh.gov.za/docs/legislation/patientsright/chartere.html.

[54] South Africa. Public Service Commission Code of conduct for public servants [homepage on the Internet]. 1997 [cited 2014 May 30]. Available from: http://www.psc.gov.za/documents/code.asp.

[55] South Africa. Department of Public Service and Administration Batho Pele handbook [homepage on the Internet]. No date [cited 2014 May 30]. Available from: http://www.dpsa.gov.za/batho-pele/docs/BP_HB_optimised.pdf.

